# Editorial: Double-edged swords: important factors connecting metabolic disorders and cancer development – from basic research to translational applications, volume II

**DOI:** 10.3389/fendo.2024.1441828

**Published:** 2024-06-19

**Authors:** Che-Pei Kung, Thibaut Barnoud, Cong-Hui Yao, Irene Bertolini, Maureen E. Murphy

**Affiliations:** ^1^ Department of Medicine, Division of Molecular Oncology, Siteman Cancer Center, Washington University School of Medicine, Saint Louis, MO, United States; ^2^ Department of Biochemistry and Molecular Biology, College of Medicine, Medical University of South Carolina, Charleston, SC, United States; ^3^ Department of Cell Biology, Blavatnik Institute, Harvard Medical School, Boston, MA, United States; ^4^ Molecular and Cellular Oncogenesis Program, The Wistar Institute, Philadelphia, PA, United States

**Keywords:** metabolic disorder, cancer, translational research, basic research, metabolism

Building upon the previous two series of articles (Double-edged Swords: Genetic Factors That Influence the Pathogenesis of Both Metabolic Disease and Cancer; Double-Edged Swords: Important Factors Connecting Metabolic Disorders and Cancer Development - From Basic Research to Translational Applications) discussing the intersections between metabolic dysfunction and cancer development, this research topic highlights new challenges and opportunities with our expanded knowledge.

Drug resistance is a significant issue in cancer therapy (1). Tirendi et al. reviewed the recent literature between 1988 and 2022 regarding therapeutic strategies and challenges of colorectal cancer (CRC) in “Colorectal cancer and therapy response: a focus on the main mechanisms involved”. The authors discussed mechanisms contributing to CRC resistance, including metabolic reprogramming in cancer stem cells. To overcome CRC resistance, metabolic adaptors such as metformin and nanoparticle-based systems have been developed to improve treatment efficacy and delivery, respectively.

In “Co-administration of MDR1 and BCRP or EGFR/PI3K inhibitors overcomes lenvatinib resistance in hepatocellular carcinoma”, Sun et al. described novel strategies to overcome resistance of hepatocellular carcinoma (HCC) to lenvatinib, a tyrosine kinase inhibitor used in patients with unresectable HCC. Following the development of lenvatinib resistance (LR), multidrug resistance protein 1 (MDR1) and breast cancer resistance protein (BCRP) transporters were upregulated, and the epidermal growth factor receptor (EGFR) and PI3K/AKT pathways were activated. As the result, combining lenvatinib with MDR1/BCRP dual inhibitor elacridar or EGFR inhibitor gefitinib proved to be effective strategies to overcome LR.

To develop novel treatments against HCC, new opportunities have been presented by our expanded understanding of the interaction between immunity and metabolism. Tu et al. described in “Hepatic macrophage mediated immune response in liver steatosis driven carcinogenesis” how liver macrophages produce inflammatory mediators to cause lipid dysfunction, steatosis and ultimately liver cancer. Treatments targeting this pathway, such as AMPK activators or dietary interventions, may be beneficial when integrated in HCC therapy.

Connections between metabolic syndrome and thyroid cancer (TC) have been described (2). In “Do metabolic factors increase the risk of thyroid cancer? a Mendelian randomization study”, Liang and Sun provided additional contexts by using Mendelian Randomization (MR) to analyze genome-wide association studies (GWAS) dataset. Their analysis revealed a protection role for high-density lipoprotein (HDL) on TC, suggesting that strategies targeting HDL regulations could have therapeutic values. Other metabolites can also be linked to TC. In “Alterations in the amino acid profile in patients with papillary thyroid carcinoma with and without Hashimoto’s thyroiditis”, Hellmann et al. used high-performance liquid chromatography-triple stage quadrupole-mass spectrometry (HPLC-TSQ-MS) to profile amino acids (AA) in the serum of patients with papillary thyroid carcinoma (PTC) with or without Hashimoto’s thyroiditis (HT). Despite sharing similar AA profiles compared with healthy controls, serum of PTC patients with HT (PTC1) can be distinguished from those without HT (PTC0) by lysine and alanine profiles, suggesting diagnostic values of AA in TC.

Some metabolites, such as 2-Hydroxyglutarate (2HG), also possess pro-tumorigenic functions (3). In “Renal oncometabolite L-2-hydroxyglutarate imposes a block in kidney tubulogenesis: Evidence for an epigenetic basis for the L-2HG-induced impairment of differentiation”, Taub et al. showed that knockdown of L-2HG dehydrogenase (L2HGDH) in Renal Proximal Tubule (RPT) cells resulted in increased 2HG level and reduced tubulogenesis by RPT cells. This result was accompanied with reduced expression of cell differentiation factors and altered methylation status of chromatin. It suggests that 2HG functions as an oncometabolite by suppressing normal differentiation.

Our understanding about the role of glucose metabolism in human diseases have spanned from diabetes to cancer (4). In “The “sweet” path to cancer: focus on cellular glucose metabolism”, Iacobini et al. reviewed the current literature contextualizing the role of aerobic glycolysis, or Warburg effect, in cancer, inflammation, and diabetes. They highlighted two important factors, the hypoxia-inducible factor-1α (HIF-1α) and M2 isoform of pyruvate kinase (PKM2), in promoting glucose metabolic rewiring to shape the immune and endocrine environments during disease progression.

Both HIF-1α and PKM2 are metabolic enzymes critical for functions in normal and cancerous cells. Mahé et al. did a deep dive, in “Genetics of enzymatic dysfunctions in metabolic disorders and cancer”, into our current knowledge about how genetic alterations in metabolic enzymes contribute to human diseases. They explored a variety of functional pathways, including the urea cycle, glycogen storage, lysosome storage, fatty acid oxidation, and mitochondrial respiration among others, that can be hijacked by dysregulation of metabolic enzymes to promote the development of metabolic disorders and cancers.

In “Is MG53 a potential therapeutic target for cancer?”, Du et al. discussed the roles MG53 plays as a target in cancer therapy. As a member of the tripartite-motif (TRIM) protein family with glucose-regulating functions, MG53 has been shown to play beneficial roles in cancer treatment. Restoring or elevating MG53 levels could enhance efficacy of chemo- and immuno-therapy while limiting associated tissue injuries. MG53’s role in metabolic regulation, however, has also been implicated in insulin resistance and cancer cachexia, leading to detrimental effects during cancer treatment.

Drug repurposing represents a promising strategy to discover novel therapies for cancer (5). In “The magic bullet: Niclosamide”, Jiang et al. reviewed the potential of niclosamide, an FDA-approved drug for tapeworm treatment, in cancer therapy considering its recently discovered ability to modify the global epigenetic landscape through metabolic reprogramming (6). With its distinctive effects on epigenetic regulation, metabolic programming, and other oncogenic and tumor suppressive mechanisms, such as Wnt/β-catenin, NF-κB, p53, and AMPK pathways, niclosamide is a promising candidate for combination therapies.

Racial disparity plays a significant role in disease progression and therapy (7). In “Population-enriched innate immune variants may identify candidate gene targets at the intersection of cancer and cardio-metabolic disease”, Yeyeodu et al. discussed the roles innate immunity and inflammation play in differential susceptibilities to metabolic disorder and cancer among racial populations. Genetic inheritances and adaptions, in response to geographically defined environmental stresses, shape the innate immune profiles in different ethnic groups. It offers important insights in development of precision therapies.

With the variety of topics covered, our discussion and learning about links between metabolic functions and cancer continue, from basic science to translational applications.

## Author contributions

C-PK: Conceptualization, Project administration, Supervision, Writing – original draft, Writing – review & editing. TB: Project administration, Writing – original draft, Writing – review & editing. C-HY: Project administration, Writing – original draft, Writing – review & editing. IB: Project administration, Writing – original draft, Writing – review & editing. MM: Project administration, Supervision, Writing – original draft, Writing – review & editing.
